# Could Computational Approaches Challenge How we Understand and Tackle Mental Disorders Stigma?

**DOI:** 10.1192/j.eurpsy.2022.198

**Published:** 2022-09-01

**Authors:** T. Gargot

**Affiliations:** Hôpital Bretonneau, Child And Adolescent Psychiatry, Tours, France

## Abstract

Most psychiatric disorders (between 50% and 90%) remain untreated although effective treatments exist (Kohn et al., 2004). Stigma is frequent toward patients and mental health professionals (Rüsch et al., 2005). It impairs access to care especially during the early signs, amplifying the treatment gap, mainly due to a shortage of resources. How the brain makes prior internal models of the world could explain why we all stigmatize. The Bayesian brain hypothesis describes the optimal combination of priors, coming from our evolution, our memory and our perceptions, to make decisions (Parr et al., 2018). This can be intuitively understood by visual illusions, but also medical categorical diagnostic reasoning (Medow, 2011). These priors allow to simplify the world and categorize people (Sherman et al., 1998). Beyond cognitive modelling, Computational approaches also led to the development of several electronic devices, from apps to social robots, (Gargot et al., 2021). Patients reported that it was easier to self-disclose toward an avatar, that cannot judge, than toward a therapist (Hang and Gratch, 2010). These technologies could improve early, scalable as well as efficient access to care (Andersson et al., 2019). Empathy is the best strategy to tackle stigma. What is it exactly? Good therapist-patient synchronization of brains (Czeszumski et al., 2020) and bodies (Ramseyer et Tschacher, 2011) could foster better communication and thus empathy. There is an urgent need to promote research in empathy (Belzung, 2017), non-specific factors in psychotherapy mechanisms (Miller et Moyers, 2021) and psychotherapy online training resources (Gargot et al., 2020).

**Disclosure:**

Thomas Gargot was paid by the French ministry of research (Doctoral school), French ministry of health (médaille d’argent, CCA-AHU) for a PhD and CCA position. During his PhD, he prepared several scientific presentations with Dr Asselborn. This work led

